# Safety, pharmacokinetics, and pharmacodynamics of SHR7280, an oral gonadotropin-releasing hormone antagonist in healthy premenopausal women

**DOI:** 10.3389/fphar.2022.1027648

**Published:** 2022-11-23

**Authors:** Yi Xu, Wei Hu, Jian Li, Xin Jiang, Ping Shi, Kai Shen, Yu Shen, Lingyu Ma, Yu Cao

**Affiliations:** ^1^ Phase I Clinical Research Center, The Affiliated Hospital of Qingdao University, Qingdao, China; ^2^ Department of Clinical Pharmacology, The Second Hospital of Anhui Medical University, Hefei, China; ^3^ Early Clinical Trial Centre, The Second Affiliated Hospital of Nanchang University, Nanchang, China; ^4^ Jiangsu Hengrui Pharmaceuticals Co., Ltd., Shanghai, China

**Keywords:** gonadotropin-releasing hormone antagonist, healthy premenopausal women, phase 1 trial, SHR7280, pharmacokinetics, pharmacodynamics, safety

## Abstract

**Background:** Treatment with gonadotropin-releasing hormone (GnRH) antagonists is a powerful strategy to suppress gonadotropin activity in women with sex hormone-dependent disorders. Herein, we provide the safety, pharmacokinetics (PK), and pharmacodynamics (PD) profiles of SHR7280, an oral non-peptide GnRH antagonist in healthy premenopausal women.

**Methods:** In this randomized, double-blinded, placebo-controlled, dose-ascending, phase 1 trial, healthy premenopausal women were randomized to receive SHR7280 or placebo orally. Four doses of SHR7280 (200, 300, 400, and 500 mg BID) were planned. Safety, PK, and PD parameters were evaluated.

**Results:** SHR7280 presented tolerable toxicity and most adverse events were mild in severity. SHR7280 showed rapid onset of action (median T_max_ ranged from 1.0 to 1.2 h for each dose), and plasma exposure was dose-dependent. PD results showed that SHR7280 300 mg BID and above suppressed estrogen concentration within the estradiol (E_2_) treatment window for endometriosis (20–50 pg/ml), inhibited the emergence of the peak of luteinizing hormone (LH) and the concentration of follicle stimulating hormone (FSH), and maintained the concentration of progesterone (P) in an anovulatory state (2 nmol/L).

**Conclusion:** SHR7280 showed favorable safety, PK, and PD profiles in the dose range of 200–500 mg BID in healthy premenopausal women. This study supports the continued clinical development of SHR7280 as a GnRH antagonist for sex hormone-dependent disorders in women.

**Clinical Trial Registration:** https://clinicaltrials.gov/ct2/show/NCT04554043, Identifier NCT04554043

## Introduction

Gonadotropin-releasing hormone (GnRH) is a vital regulator on the hypothalamus-pituitary-gonadal reproductive axis (Valenta and Zolman, 1977; Metallinou et al., 2007). Natural GnRH is a decapeptide hormone synthesized by the arcuate nucleus of the hypothalamus. It is secreted in pulse form through the pituitary portal system to stimulate the pituitary gland to synthesize and secrete gonadotropins, including luteinizing hormone (LH) and follicle stimulating hormone (FSH), to promote the secretion of estradiol (E_2_, the main estrogen) and progesterone by the ovary and testosterone by testes, which play physiological roles in the reproductive system, cardiovascular system, and musculoskeletal system.

Therapies targeting GnRH have been widely used to treat or alleviate a variety of sex hormone-dependent diseases, such as endometriosis, uterine fibroid, polycystic ovary syndrome, precocious puberty, as well as in other therapeutic areas, such as suppression of the LH peak during assisted reproduction treatment and prevention of ovarian failure caused by systemic chemotherapy (Casper, 1991; Conn and Crowley, 1991; Chen et al., 2019). The use of GnRH agonists for 10–15 days could desensitize the GnRH receptor in the pituitary, decrease the level of FSH and LH, and finally result in a reduction in gonadal synthesis and secretion of sex hormones to achieve menopause or castration in women (Ortmann et al., 2002). However, GnRH agonists are commonly associated with a slow onset of action, temporary worsening symptoms (flare-up effect), 2–3 weeks of GnRH receptor desensitization, and severe hypoestrogenic side effects. These characteristics limit the use of GnRH agonists as long-term effective and safe drugs for the treatment of sex hormone-related diseases.

GnRH antagonists are commonly administered orally and show rapid onset of action, and do not have flare-up effect, overcoming several shortcomings of GnRH agonists and expanding the current treatment options (Blumenfeld, 2001; Huirne and Lambalk, 2001; Betz et al., 2008). In recent years, non-peptide oral GnRH antagonists have been developed as a promising treatment strategy for this population (Bouchard and Fauser, 2000; Huirne and Lambalk, 2001; Betz et al., 2008). Elagolix, an oral GnRH antagonist, has been approved by the US Food and Drug administration (FDA) to treat moderate to severe pain associated with endometriosis and is currently being developed to treat severe menstrual bleeding associated with hysteromyoma (Archer et al., 2017; Diamond et al., 2017; Taylor et al., 2017; Surrey et al., 2018; Schiffman et al., 2020; Schlaff et al., 2020). It can profoundly inhibit the secretion of ovarian sex steroids, which is similar to the effects of ovariectomy. However, in several countries or regions including China, elagolix has not been approved for use in women with sex hormone-related disease. Therefore, novel treatment options are needed in this population.

SHR7280, a non-peptide, direct, and competitive GnRH antagonist, blocks the binding of endogenous GnRH to its receptor, inhibits the synthesis and release of gonadotropins such as LH and FSH, and reduces testosterone and estradiol levels. *In vitro* studies have shown that SHR7280 had a high oral bioavailability (63.1% in rats and 64.5% in dogs), a short half-life (1.0–2.2 h in rats and 3.2–4.5 h in dogs), similar drug exposures between males and females, and marginal inhibition on CYP450 enzyme. SHR7280 has good transmembrane ability, equivalent to the medium to upper level of human intestinal absorption (50%–70%). It presents fewer cardiovascular, respiratory, and nervous systems side effects and showed no genotoxicity in animal models (data on file, Hengrui). SHR7280 is currently under clinical development for women with endometriosis or uterine fibroids. Herein, we report the results of a phase 1 study, in which the tolerability, safety, pharmacokinetics (PK), and pharmacodynamics (PD) of SHR7280 were evaluated in healthy premenopausal women.

## Materials and methods

### Study design and participants

This randomized, double-blind, placebo-controlled, dose-ascending, phase 1 trial of SHR7280 (clinicaltrials.gov, NCT04554043) involving healthy premenopausal women was conducted in three study centers (Supplementary Table S1).

Eligible participants were adult premenopausal women with regular menstrual cycles of 24–32 days and menstruation of 3–7 days per month for at least 3 months, between 18 and 45 years of age, with a body mass index of 18–30 kg/m^2^. The main exclusion criteria were pregnancy or breastfeeding; FSH ≥25 mIU/ml; abnormal uterine bleeding within 3 months; positive for hepatitis B virus, hepatitis C virus, human immunodeficiency virus antibody, or *Treponema pallidum*; use of GnRH agonists within 6 months before screening, and use of GnRH antagonists or any sex hormone drugs within 2 months before screening; had chronic or serious diseases affecting drug absorption, distribution, metabolism, and excretion; and history of drugs to inhibit or induce liver drug metabolism in the liver 1 month before drug administration.

Eligible women received oral SHR7280 tablets or placebo twice daily for 21 consecutive days. The treatment dose started at 200 mg BID and then increased to 300, 400, and 500 mg BID. The starting dose, dosing frequency, and maximum dose were based on the PD data and tolerability of a phase 1 trial of SHR7280 in healthy women (registered at http://www.chictr.org.cn/, CTR20181472). In each dose cohort, 12 participants were randomized in a 5:1 ratio to receive SHR7280 (*n* = 10) or placebo (*n* = 2). The dose escalation was performed based on safety assessment after 50% of participants in the preceding lower-dose group completed safety follow-up on day 23 or 2 days after the last dose by the Safety Review Committee. Dosing for each participant began within 2–4 days after the onset of menstruation.

### Safety assessment

The safety evaluation included adverse events (AEs), menstruation, vital signs, physical examination, laboratory examination, 12-lead ECG, and breast B-ultrasound. The assessments were performed from the first dose to day 49 (±2) or 28 (±2) days after the last dose. AEs were classified according to the Medical Dictionary for Regulatory Activities (MedDRA, v24.0) and AE severity was classified according to the National Cancer Institute’s Common Terminology Criteria for Adverse Events (NCI-CTCAE, v5.0). Grade 1 AE was defined as a mild event, grade 2 AE was defined as a moderate event, and grade 3 or higher grade 1 AE and grade 2 AE was defined as a severe event.

### Pharmacokinetic and pharmacodynamic analysis

The time points of blood sample collection used for PK analysis included: predose (0 h), 0.5, 1, 1.5, 2, 4, 6, 8, 10, 12, and 16 h on day 1, predose on days 2, 3, 5, 7, 9, 11, 13, 17, and 19, and predose, 0.5, 1, 1.5, 2, 4, 6, 8, 10, 12, 16, 24, 36, and 48 h on day 21. Three milliliters of venous blood was collected at each time point. The PK parameters of SHR7280 were measured using high-performance liquid chromatography tandem mass spectrometry (HPLC-MS-MS) and analyzed by Frontage Laboratories Co. Ltd. (Shanghai, China). The lower limit of quantification (LLOQ) for SHR7280 was 2 ng/ml.

Blood samples for PD analysis were collected at following time points, including predose (0 h), 2, 4, 6, 8, 10, 12, and 16 h on day 1, predose on days 2, 3, 5, 7, 9, 11, 13, 15, 17, and 19, and predose, 2, 4, 6, 8, 10, 12, 16, 24, 36, and 48 h on day 21. Approximately 3.5 ml of venous blood was collected at each time point and serum was separated for serum hormone evaluations. PD parameter concentrations were detected using the chemiluminescence method on the Architect iSR2000 Immunoassay analyzer (Abbott Laboratories, Abbott Park, IL, United States) and Alinity analyzer (Abbott Laboratories, Abbott Park, IL, United States) by KingMed Diagnostics Group Co., Ltd. (Guangzhou, China). The LLOQ values for E_2_, progesterone, FSH, and LH with Architect iSR2000 Immunoassay analyzer were 13 pg/ml, 0.35 nmol/L, 0.75 mIU/ml, and 0.11 mIU/ml, respectively, and with Alinity analyzer were 10 pg/ml, 0.32 nmol/L, 0.05 mIU/ml, and 0.09 mIU/ml, respectively.

## Outcomes

The primary endpoint was safety. Secondary endpoints were PK and PD results. The PK parameters included time to maximum plasma concentration (T_max_), maximum plasma concentration (C_max_), and area under the plasma concentration-time profile (AUC) on day 1 and half-life (t_1/2_), AUC, apparent volume of distribution (V_z_/F), apparent total clearance (CL/F), C_max_, T_max_, plasma trough concentration (C_trough_), and accumulation ratio (R_acc_) on day 21. The PD parameters included E_2_, LH, FSH, and progesterone.

### Statistical analysis

The sample size of this study was determined according to the policy of the China National Medical Products Administration on the clinical pharmacokinetics of chemical drugs and the recommendation of a previous study (Shen et al., 2019), and the statistical assumptions of the sample size were not calculated.

The AUCs of the PK and PD parameters were calculated in a noncompartmental model using Phoenix WinNonlin v8.2 or higher. The ANOVA model was performed to analyze the correlation of the dose and standardized PK parameters after logarithmic transformation, estimate the geometric ratio of the least squares mean, and the corresponding 90% confidence interval (CI). The correlations between PK and PD were evaluated using the E_max_ model. The safety data are presented descriptively. Statistical analyses were performed using SAS v9.4 (SAS Institute, Inc., Cary, NC, United States).

## Results

### Patients

**FIGURE 1:**
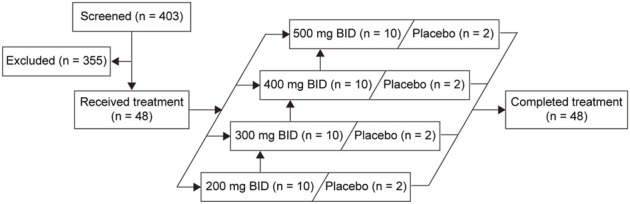
Volunteer disposition.

A total of 48 eligible participants were enrolled and randomly assigned. Forty participants received the SHR7280 (10 participants per dose) and eight received placebo (Figure 1). One participant in the 500 mg BID group discontinued treatment due to an AE on day 21; the other 47 participants completed study drug administrations for 21 consecutive days. The baseline characteristics of the participants in different dose groups of SHR7280 and the placebo group were generally similar (Table 1).

**TABLE 1:** Baseline characteristics.

	200 mg BID (*n* = 10)	300 mg BID (*n* = 10)	400 mg BID (*n* = 10)	500 mg BID (*n* = 10)	SHR7280 total(*n* = 40)	Placebo (*n* = 8)
Age, years	32.7 ± 9.5	30.7 ± 9.3	32.7 ± 6.5	34.1 ± 5.5	32.6 ± 7.7	35.9 ± 3.4
BMI, kg/m^2^	22.8 ± 2.4	23.0 ± 3.1	22.4 ± 3.4	24.0 ± 1.7	23.1 ± 2.7	24.0 ± 2.9
Height, cm	160.2 ± 6.9	159.3 ± 6.3	160.8 ± 4.0	160.9 ± 5.4	160.3 ± 5.6	159.4 ± 7.4
Weight, kg	58.7 ± 8.8	58.3 ± 6.8	57.9 ± 9.6	62.1 ± 4.1	59.3 ± 7.5	60.8 ± 7.2

Data are mean ± SD.

## Safety

Safety was assessed in the 48 participants who received at least one dose of SHR7280 or placebo. The proportion of participants who experienced at least one AE was 97.5% (39/40) in the SHR7280 group and 87.5% (7/8) in the placebo group (Table 2). The most common AEs that occurred in the SHR7280 group were oligomenorrhoea (95.0% [38/40] with SHR7280 vs. 0 with placebo), increased alanine aminotransferase (20.0% [8/40] vs. 25.0% [2/8]), and positive urine leukocyte esterase (20.0% [8/40] vs. 25.0% [2/8]). The majority of AEs were mild events. Moderate AEs were reported in 17.5% (7/40) of the participants with SHR7280 and none with placebo. No severe AEs were reported in either group. Both the incidence and the severity of AEs were not proportional to dose between the four SHR7280 dose groups. There were no deaths or serious AEs.

**TABLE 2:** AEs.

	200 mg BID (*n* = 10)	300 mg BID (*n* = 10)	400 mg BID (*n* = 10)	500 mg BID (*n* = 10)	SHR7280 total (n = 40)	Placebo (*n* = 8)
Any AE	9 (90.0)	10 (100)	10 (100)	10 (100)	39 (97.5)	7 (87.5)
Reproductive system and breast disorders						
Oligomenorrhoea	8 (80.0)	10 (100)	10 (100)	10 (100)	38 (95.0)	0
Heavy menstrual bleeding	1 (10.0)	2 (20.0)	0	1 (10.0)	4 (10.0)	0
Investigations						
Alanine aminotransferase increased	2 (20.0)	3 (30.0)	0	3 (30.0)	8 (20.0)	2 (25.0)
Urine leukocyte esterase positive	2 (20.0)	2 (20.0)	2 (20.0)	2 (20.0)	8 (20.0)	2 (25.0)
Neutrophil count decreased	0	2 (20.0)	3 (30.0)	2 (20.0)	7 (17.5)	0
Blood thyroid stimulating hormone increased	1 (10.0)	1 (10.0)	4 (40.0)	0	6 (15.0)	0
White blood cell count decreased	0	1 (10.0)	2 (20.0)	2 (20.0)	5 (12.5)	0
White blood cells urine positive	1 (10.0)	1 (10.0)	2 (20.0)	0	4 (10.0)	1 (12.5)
Estradiol decreased	2 (20.0)	1 (10.0)	0	1 (10.0)	4 (10.0)	0
Thyroxine free decreased	2 (20.0)	1 (10.0)	0	1 (10.0)	4 (10.0)	0
Gastrointestinal disorders						
Abdominal pain	2 (20.0)	2 (20.0)	1 (10.0)	0	5 (12.5)	4 (50.0)
Diarrhea	2 (20.0)	1 (10.0)	1 (10.0)	0	4 (10.0)	1 (12.5)
Infections and infestations						
Bacterial vulvovaginitis	0	3 (30.0)	0	2 (20.0)	5 (12.5)	0
Asymptomatic bacteriuria	2 (20.0)	2 (20.0)	0	0	4 (10.0)	3 (37.5)
Blood and lymphatic system disorders						
Anemia	1 (10.0)	1 (10.0)	2 (20.0)	0	4 (10.0)	0

Data are n (%). AEs occurred in ≥10% of total participants who received SHR7280 are listed.

The incidence of treatment-related AEs was 97.5% (39/40) and 87.5% (7/8) in participants receiving SHR7280 and placebo, respectively (Supplementary Table S2). In participants who received SHR7280, the most common treatment-related AEs were oligomenorrhoea (95.0% [38/40] with SHR7280 vs. 0 with placebo), increased alanine aminotransferase (20.0% [8/40] vs. 25.0% [2/8]), and increased blood thyroid stimulating hormone (15.0% [6/40] vs. 0).

One participant in the SHR7280 500 mg BID group discontinued treatment due to AE. The biochemical blood test on day 21 showed that she had abnormal hepatic function, but no gastrointestinal tract symptoms were observed. This AE was judged as a moderate event and related to SHR7280. She stopped the second administration on day 21, took dicyclohol, diammonium glycyrrhizinate, glutathione, and liver-protecting drugs orally, and finally regressed later after 4 weeks of treatment.

### Pharmacokinetics profile

The 40 participants who received at least one dose of SHR7280 had at least one qualified blood sample for plasma drug concentration and evaluation of PK parameters, and were included in the PK analysis.

**FIGURE 2:**
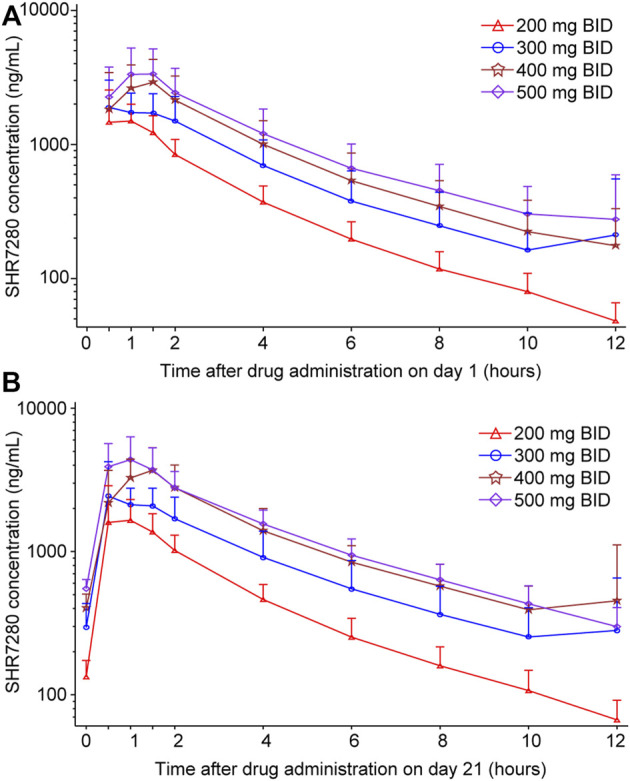
Plasma concentration-time profiles of SHR7280 on day 1 **(A)** and day 21 **(B)**. Data are presented as mean (±SD).

After a single oral administration on day 1, SHR7280 was rapidly absorbed, with a similar T_max_ (1.0 h, 1.0 h, 1.2 h, and 1.0 h) to reach the concentration peaks at the dose of 200, 300, 400, and 500 mg BID (Table 3). The mean C_max_ of plasma SHR7280 was 1890 ± 836 (44.3) [mean ± SD (%CV)], 2610 ± 950 (36.4), 3680 ± 1380 (37.5), and 4070 ± 1770 (43.4) ng/ml in the four dose groups, respectively. Plasma exposure of SHR7280 (C_max_ and AUC_0-12h_) increased with increasing doses of SHR7280 within the range of 200–500 mg BID (Figure 2).

**TABLE 3:** PK parameters.

		200 mg BID (*n* = 10)	300 mg BID (*n* = 10)	400 mg BID (*n* = 10)	500 mg BID (*n* = 10)
Day 1					
T_max_, h	Median (range)	1.0 (0.5–1.5)	1.0 (0.5–2.0)	1.2 (0.5–1.5)	1.0 (0.5–1.5)
C_max_, ng/mL	Mean ± SD (%CV)	1890 ± 836 (44.3)	2610 ± 950 (36.4)	3680 ± 1380 (37.5)	4070 ± 1770 (43.4)
	GeoMean (%GeoCV)	1680 (63.1)	2440 (41.8)	3120 (92.9)	3570 (69.1)
AUC_0-12h_, h*ng/mL	Mean ± SD (%CV)	4610 ± 1530 (33.2)	7520 ± 2910 (38.7)	10500 ± 4300 (41.1)	12800 ± 6120 (47.9)
	GeoMean (%GeoCV)	4270 (50.0)	7100 (35.6)	9280 (67.0)	10900 (75.8)
Day 21					
T_max_, h	Median (range)	1.0 (0.5–1.9)	1.0 (0.5–1.5)	1.5 (0.5–2.0)	1.0 (0.5–2.0)
C_max_, ng/mL	Mean ± SD (%CV)	2140 ± 921 (43.1)	3050 ± 1480 (48.6)	4200 ± 1470 (34.9)	4960 ± 1810 (36.5)
	GeoMean (%GeoCV)	1910 (61.3)	2770 (47.7)	3840 (55.0)	4560 (51.5)
AUC_0-12h_, h*ng/mL	Mean ± SD (%CV)	5510 ± 1800 (32.7)	9780 ± 3750 (38.3)	14700 ± 5010 (34.0)	16800 ± 4900 (29.2)
	GeoMean (%GeoCV)	5180 (42.3)	9180 (38.7)	13600 (50.9)	16000 (36.5)
t_1/2_, h	Mean ± SD (%CV)	3.2 ± 0.6 (19.3)	3.5 ± 0.6 (16.7)	3.3 ± 0.9 (26.9)	3.3 ± 0.5 (16.2)
	GeoMean (%GeoCV)	3.14 (18.6)	3.48 (17.0)	3.26 (21.8)	3.30 (15.9)
V_z_/F, L	Mean ± SD (%CV)	191 ± 93 (48.7)	174 ± 64.6 (37.2)	158 ± 111 (69.9)	156 ± 55.7 (35.8)
	GeoMean (%GeoCV)	175 (44.3)	164 (36.2)	138 (51.6)	149 (30.9)
CL/F, L/h	Mean ± SD (%CV)	42.2 ± 23.1 (54.7)	34.8 ± 13.4 (38.4)	33.7 ± 24.4 (72.3)	33.4 ± 15.1 (45.3)
	GeoMean (%GeoCV)	38.6 (42.3)	32.7 (38.7)	29.4 (50.9)	31.2 (36.5)
C_trough_, ng/mL	Mean ± SD (%CV)	73.3 ± 36.3 (49.6)	171 ± 107 (63.0)	258 ± 128 (49.6)	299 ± 108 (36.0)
	GeoMean (%GeoCV)	66.1 (49.9)	146 (62.1)	229 (58.0)	280 (40.7)
R_acc_, h	Mean ± SD (%CV)	1.2 ± 0.2 (15.4)	1.3 ± 0.3 (22.2)	1.5 ± 0.4 (24.6)	1.6 ± 0.7 (45.0)
	GeoMean (%GeoCV)	1.21 (15.8)	1.29 (30.1)	1.46 (24.9)	1.46 (43.1)

Data are mean ± SD, unless otherwise specified.

On day 21 (steady state), the median time to reach concentration peaks was 1.0–1.5 h, and the elimination half-life (t_1/2_) was 3.2–3.5 h. Both T_max_ and t_1/2_ were similar across the four dose groups. Drug exposure (C_max_ and AUC_0-12h_) on day 21 in each dose group was also dose-dependent, and was similar to the pattern observed on day 1. The mean CL/F and V_z_/F values ranged from 33.4 to 42.2 L/h and 156–191 L, respectively. Little or no accumulation was observed, because the mean R_acc_ for the AUC of each group was 1.2 ± 0.2 (15.4) [mean ± SD (%CV)], 1.3 ± 0.3 (22.2), 1.5 ± 0.4 (24.6), and 1.6 ± 0.7 (45.0), respectively.

The ANOVA model was used to analyze the correlation of drug exposure with dose. The results showed that both dose-normalized C_max_ and dose-normalized AUC_0-12h_ were generally similar between different dose groups (Supplementary Figure S1; Supplementary Table S3), further confirming the observed dose-dependent plasma exposure of SHR7280 within the range of 200–500 mg BID.

### Pharmacodynamics profile

PD was assessed in all 48 participants who received at least one dose of SHR7280 or placebo and had at least one qualified sample for the assessment of PD. The mean AUC_0-21d_ values of E_2_, progesterone, LH, and FSH in the SHR7280 groups had an apparent decrease compared to the placebo group and gradually declined or tended to stabilize with increasing SHR7280 dose (Supplementary Table S4).

**FIGURE 3:**
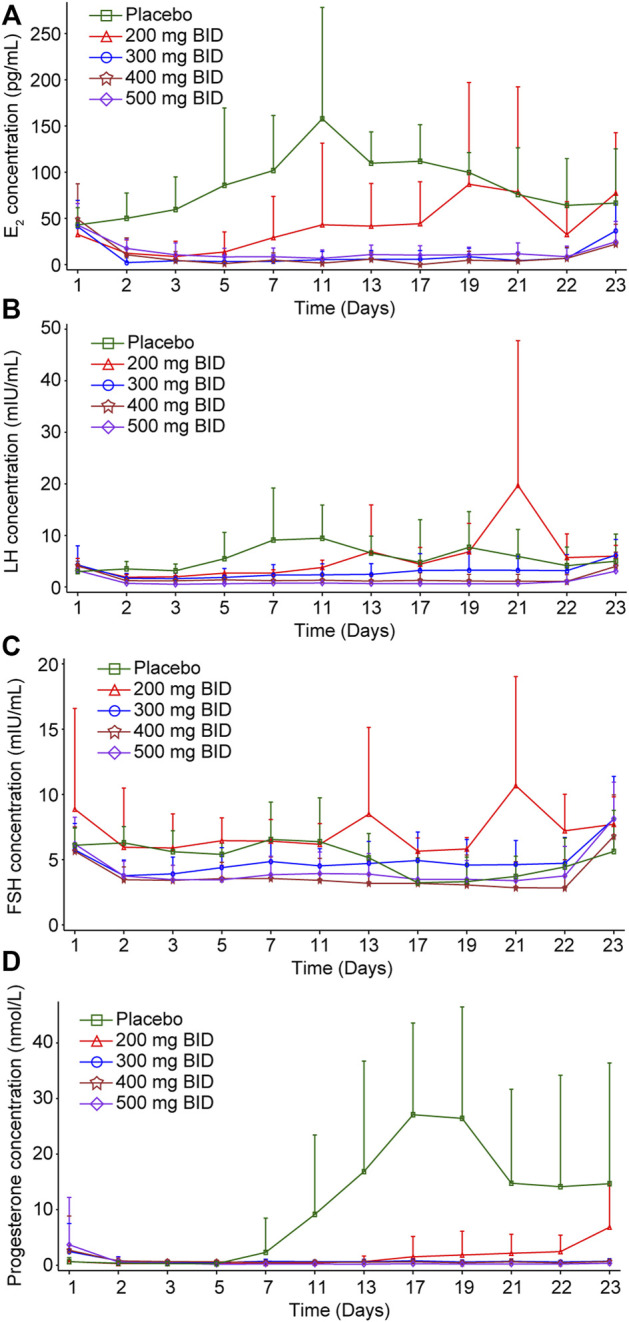
Inhibition effects of increasing doses of SHR7280 on E_2_ **(A)**, LH **(B)**, FSH **(C)**, and progesterone **(D)**.

Compared to the placebo group, the suppression of E_2_ concentration by SHR7280 was dose-dependent (Figure 3A). The E_2_ concentration decreased rapidly after SHR7280 administration within hours. Administration of 200 mg BID could effectively inhibit the level of E_2_ from day 2 to day 5, but E_2_ levels began to rebound from day 5. The dose of 300 mg BID and above maintained the estrogen concentration within the estradiol treatment window for endometriosis (20–50 pg/ml) from day 2 to day 21 without any rebound (Barbieri, 1992). The inhibitory effects of 300, 400, and 500 mg BID were similar, indicating that SHR7280 at 300 mg BID may have reached inhibition saturation of E_2_.

The progesterone level decreased rapidly after administration (Figure 3B). A 200 mg BID dose of SHR7280 showed partial suppression of progesterone concentrations and progesterone levels rebounded from day 17. The mean value of progesterone in the 300 mg BID and above dose groups from day 2 to day 21 was maintained lower than 2 nmol/L, which was considered as the progesterone concentration in the anovulatory state (Ng et al., 2017). Similar inhibition effects of 300, 400, and 500 mg BID also indicated that the administration of 300 mg BID of SHR7280 may have reached saturation inhibition of progesterone.

LH concentration in the SHR7280 groups decreased immediately after administration compared to the placebo group (Figure 3C). Throughout the treatment period, there was no LH peak in the 300 mg BID and above dose groups, and the LH curve was stable without fluctuation, suggesting that ovulation could not occur. Inhibition of LH also reached saturation at a dose of 300 mg BID and above.

The FSH level gradually decreased after SHR7280 administration (Figure 3D). Compared to placebo, the FSH concentrations in participants receiving 300 mg BID and higher doses of SHR7280 were generally lower during the treatment period.

### Pharmacokinetic and pharmacodynamic correlations

The E_max_ model with a non-linear regression fitting was used to explore the correlation between exposure and PD parameters. With increasing drug exposure (AUC_0-21d_), the inhibitory effects of SHR7280 on sex hormones levels increased and gradually approached saturation at higher doses (Supplementary Figure S2). The maximum inhibition (I_max_) for E_2_, progesterone, LH, and FSH was 32209.41 h*pg/mL, 640.11 h*nmol/L, 5907.08 h*mIU/mL, and 5401.48 h*mIU/mL, respectively, and the IC_50_ was 8652.04 h*ng/mL, 22872.27 h*ng/mL, 18038.48 h*ng/mL, and 28885.94 h*ng/mL, respectively.

## Discussion

This phase 1 study reported the PK, PD, and safety of the GnRH antagonist SHR7280 in healthy premenopausal women. PK results showed a rapid increase in plasma levels of SHR7280 in a dose-dependent manner within the range of 200–500 mg BID. Little or no accumulation was observed after multiple administrations for 21 days. The PD results demonstrated the effective inhibition of SHR7280 on sex hormones including E_2_, progesterone, LH, and FSH, which was also dose-dependent. Administration of 300 mg BID or more showed maximum repression of sex hormone levels and could reach inhibition saturation. Safety results showed good tolerability of SHR7280; most AEs were mild events.

The overall incidence and severity of AEs in the SHR7280 group were similar to those in the placebo group. The most common AE in the SHR7280 group was oligomenorrhoea (95.0%, 38/40), while none of the participants in the placebo group experienced oligomenorrhoea. SHR7280 has been reported to inhibit the hypothalamic-pituitary-ovarian (HPO) axis and ovulation, thus affecting the menstrual cycle during the treatment period, and all participants with oligomenorrhoea returned to normal menstruation within 45 days after discontinuation of SHR7280. Therefore, we believe that oligomenorrhoea was an expected safety signal and that any AEs experienced by the participants were temporary and mild.

One participant in the 500 mg BID dose group discontinued the last dose of study drug on the last day of administration due to abnormal hepatic function of moderate severity. For the other participants the hepatic function was normal as revealed by laboratory tests during this study, and there were no abnormal hepatic symptoms such as jaundice, nausea, vomiting, or liver pain, although attention should be paid to the potential hepatic dysfunction associated with SHR7280 in future studies. The incidence of bacterial vulvovaginitis and vulvovaginal candidiasis in the SHR7280 group was 12.5% (5/40) and 7.5% (3/40), respectively, while the incidences of the two AEs in the placebo group were 0. These AEs may be related to the low estrogen status of participants after SHR7280 administration. The low sex hormone status led to a decline in local immune capacity of the reproductive system, which made the participants more vulnerable to pathogens. Therefore, in future studies of long-term SHR7280 treatment, the inflammatory response of the reproductive system of participants also deserves closer attention. Furthermore, hot flashes, which have been reported in studies of elagolix (Struthers et al., 2009), are related to the deep inhibition of estrogen and very common in the use of GnRH inhibitors. However, in our SHR7280 study, no hot flashes were observed, which warrants further investigation on the long-term use of SHR7280.

SHR7280 was rapidly released in the plasma after oral administration, with a median T_max_ of 1.0–1.5 h within the dose range of 200–500 mg BID, which was similar to that observed with elagolix treatment. SHR7280 showed little or no accumulation in plasma after repeat administration. Plasma exposure of SHR7280 at the same dose was approximately three times higher than that of elagolix in the dose range of 200–400 mg BID. Elimination of SHR7280 was more rapid than that of elagolix (Struthers et al., 2009; Ng et al., 2017; Winzenborg et al., 2018).

The PD findings of SHR7280 in this study were similar to those of elagolix (Struthers et al., 2009; Ng et al., 2017; Winzenborg et al., 2018). SHR7280 effects on sex hormone levels were effective and dose-dependent. Inhibition of E_2_, progesterone, LH, and FSH by SHR7280 was synchronized. Within the dose range of 300–500 mg BID, the regulation of E_2_, progesterone, LH, and FSH by SHR7280 reached maximum inhibition and inhibition saturation, providing evidence of the recommended dose for further phase 2 study in patients with uterine fibroid related bleeding or endometriosis-associated pain.

The main limitation of this study was the small sample size, which may have introduced bias in the final results. Additionally, the follow-up time for this study was short and some AEs caused by long-term medications may have been missed. Although the objective of this phase 1 study was to investigate the safety profile of SHR7280, the PK and PD properties of SHR7280 support the further clinical development of this drug. These results should be validated in future clinical trials.

In conclusion, SHR7280 was well tolerated within the 21 consecutive days of administration. SHR7280 showed rapid onset of action and effective suppression of sex hormone levels. This phase 1 study in healthy premenopausal women supported its further clinical development as a GnRH antagonist for sex hormone-dependent disorders in women.

## Data Availability

The original contributions presented in the study are included in the article/Supplementary Material, further inquiries can be directed to the corresponding author.
