# Supplementary material for: Safety, pharmacokinetics, and pharmacodynamics of SHR7280, an oral gonadotropin-releasing hormone antagonist in healthy premenopausal women
Source: Front Pharmacol. 2022 Nov 23;13:1027648. doi: 10.3389/fphar.2022.1027648 (PMC9727091; doi:10.3389/fphar.2022.1027648)
Supplement: Supplementary file 1 [file DataSheet1.PDF]

Supplement

Supplement to: Yi Xu, et al. Safety, pharmacokinetics, and pharmacodynamics of SHR7280, an oral gonadotropin-releasing hormone antagonist in healthy premenopausal women

Table of contents

Figure S1. Box-whisker plots of dose-normalized exposure of SHR7280 on day 21 .. 2

Figure S2. E_{max} model demonstrated the correlations between SHR7280 exposure (AUC_{0-21d}) on day 21 and PD parameters 3

Table S1. Participating sites 4

Table S2. Treatment-related AEs 5

Table S3. ANOVA on the logarithm of selected PK parameters of SHR7280 in plasma 6

Table S4. AUC_{0-21d} of PD parameters of SHR7280 7

Figure S1. Box-whisker plots of dose-normalized exposure of SHR7280 on day 21

(A) Dose-normalized C_{max}. (B) Dose-normalized AUC_{0-12h}.

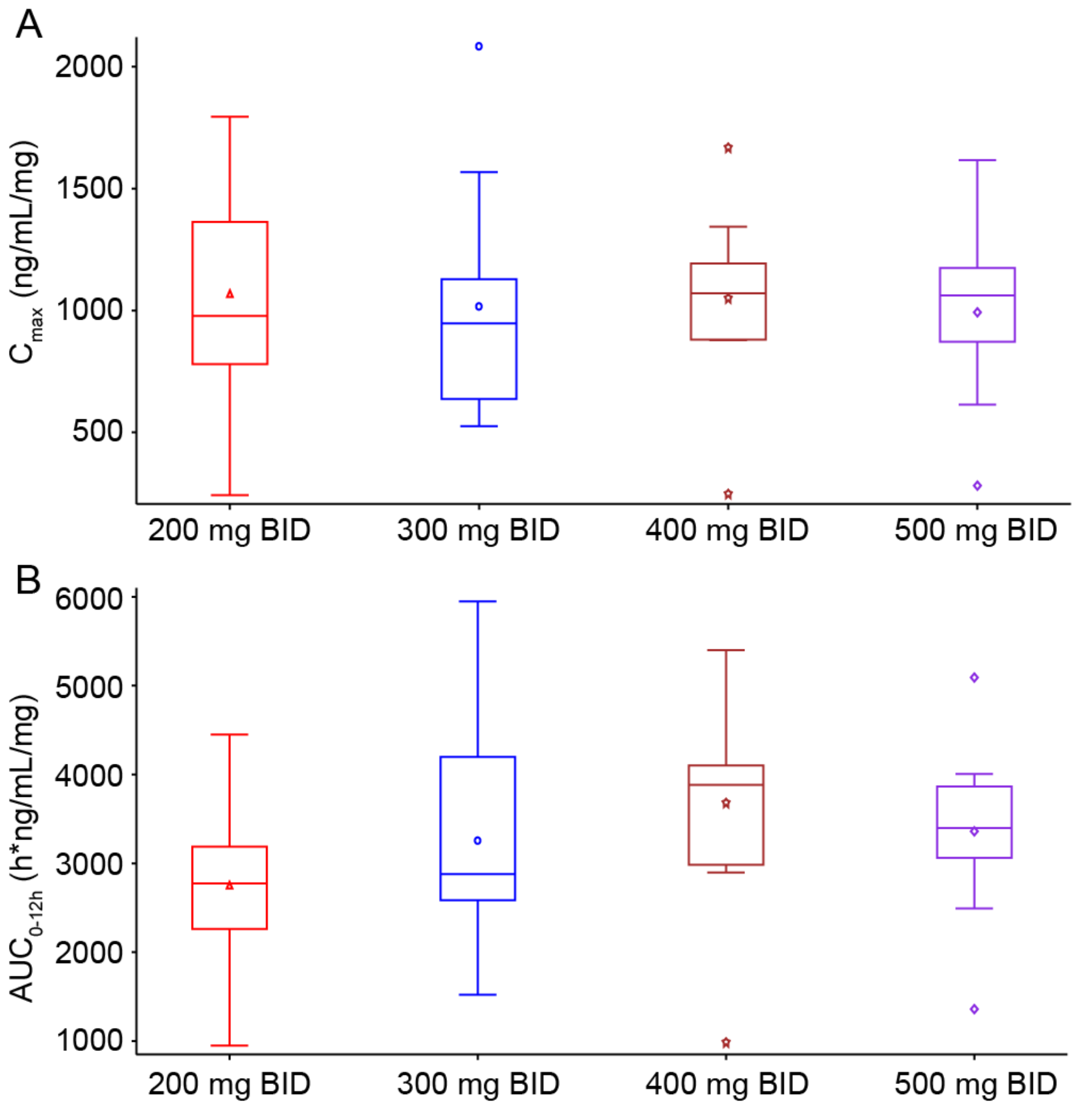

Figure S2. The E_{max} model demonstrated the correlations between SHR7280 exposure (AUC_{0-21d}) on day 21 and PD parameters

(A) E_2. (B) Progesterone. (C) LH. (D) FSH.

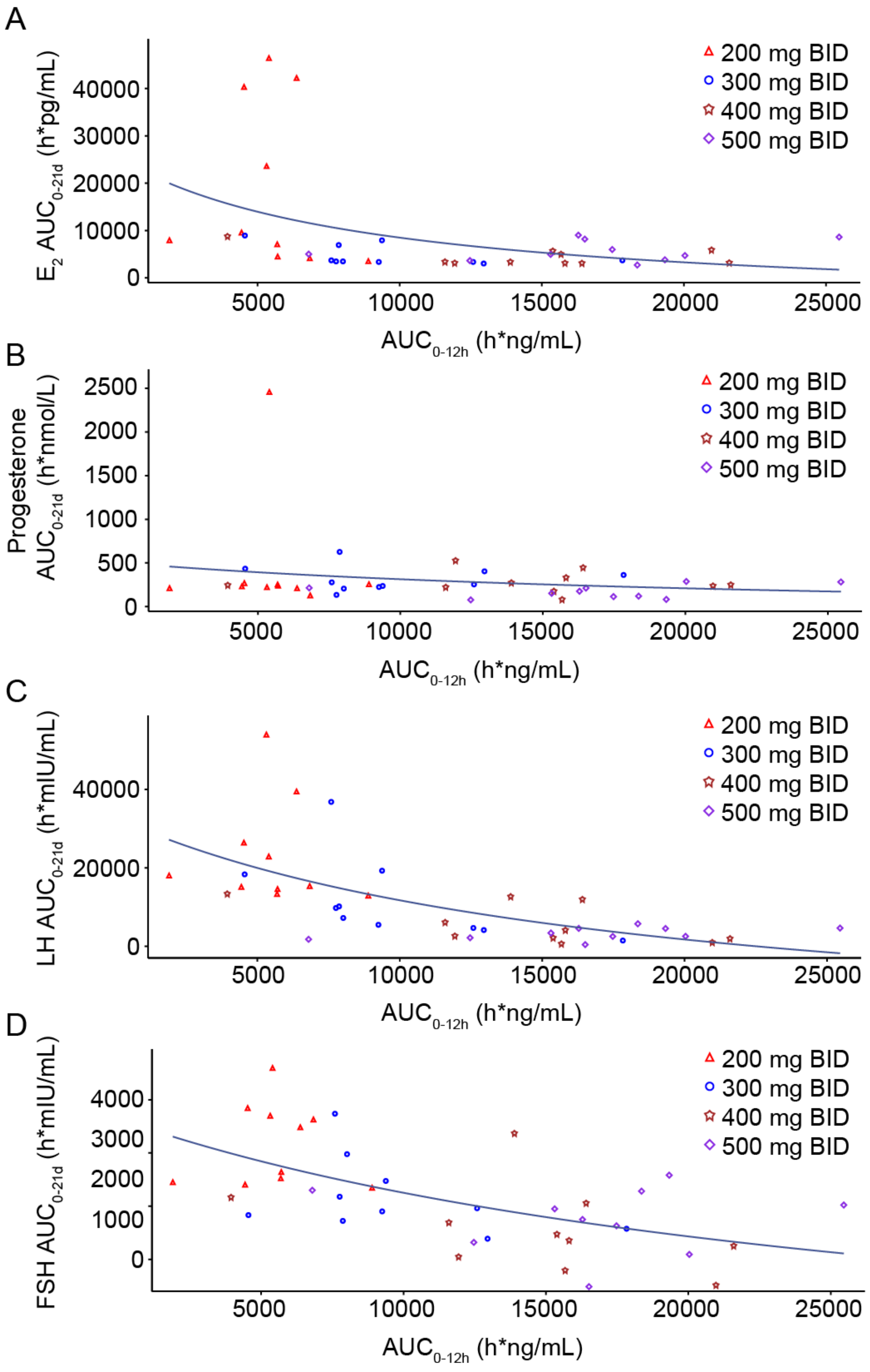

Table S1. Participating sites

Investigators	Participating sites	Number of participants
Wei Hu	The Second Affiliated Hospital of Anhui Medical University	26
Yu Cao	The Affiliated Hospital of Qingdao University	19
Jian Li	The Second Affiliated Hospital of Nanchang University	3

Table S2. Treatment-related AEs

	**SHR7280 total (n=40)**	**Placebo (n=8)**
Any treatment-related AE	39 (97.5)	7 (87.5)
Oligomenorrhoea	38 (95.0)	0
Alanine aminotransferase increased	8 (20.0)	2 (25.0)
Blood thyroid stimulating hormone increased	6 (15.0)	0
Abdominal pain	5 (12.5)	4 (50.0)
Asymptomatic bacteriuria	4 (10.0)	3 (37.5)
Diarrhea	4 (10.0)	1 (12.5)
Heavy menstrual bleeding	4 (10.0)	0
Estradiol decreased	4 (10.0)	0
Thyroxine free decreased	4 (10.0)	0
Mouth ulceration	3 (7.5)	1 (12.5)
Vaginal hemorrhage	3 (7.5)	0
Neutrophil count decreased	3 (7.5)	0
Constipation	3 (7.5)	0
Bacterial vulvovaginitis	3 (7.5)	0
Vulvovaginal candidiasis	3 (7.5)	0
Dysmenorrhoea	2 (5.0)	0
Vaginal discharge	2 (5.0)	0
Polymenorrhoea	2 (5.0)	0
White blood cell count decreased	2 (5.0)	0
White blood cells urine positive	2 (5.0)	0
Aspartate aminotransferase increased	2 (5.0)	0
Anaemia	2 (5.0)	0
Abdominal distension	1 (2.5)	2 (25.0)
Fibrin D dimer increased	1 (2.5)	1 (12.5)
Blood bilirubin increased	1 (2.5)	1 (12.5)
Cough	1 (2.5)	1 (12.5)
Blood triglycerides increased	0	1 (12.5)
Palpitations	0	1 (12.5)
Chest discomfort	0	1 (12.5)

Data are n (%). Treatment-related AEs occurred in ≥5% of participants in either group are listed. Events are shown in descending order of frequency in the SHR7280 group.

Table S3. ANOVA on the logarithm of selected PK parameters of SHR7280 in plasma

	**200 mg BID (n=10)**	**300 mg BID (n=10)**	**400 mg BID (n=10)**	**500 mg BID (n=10)**
**C_{max}, ng/mL**				
Least squares mean (90% CI)	1070 (842-1300)	1020 (790-1240)	1050 (823-1280)	992 (766-1220)
Least squares geomean (90% CI)	953 (728-1250)	924 (705-1210)	961 (734-1260)	911 (696-1190)
Ratio of least squares geomean, % (90% CI) *		96.9 (66.2-142)	101 (68.8-148)	95.6 (65.3-140)
**$AUC_{0-12h,ss}$, h*ng/mL**				
Least squares mean (90% CI)	2750 (2160-3350)	3260 (2670-3850)	3680 (3090-4270)	3360 (2770-3950)
Least squares geomean (90% CI)	2590 (2080-3210)	3060 (2460-3800)	3400 (2740-4220)	3200 (2580-3980)
Ratio of least squares geomean, % (90% CI) *		118 (87.1-161)	131 (96.6-178)	124 (91.0-168)

* Relative to 200 mg BID.

Table S4. $AUC_{0\text{-}21d}$ of PD parameters of SHR7280

	**200 mg BID (n=10)**	**300 mg BID (n=10)**	**400 mg BID (n=10)**	**500 mg BID (n=10)**	**Placebo (n=8)**
$AUC_{0\text{-}21d}$ of E_2, h*pg/mL	18900 ± 17600	4790 ± 2230	4470 ± 1880	5680 ± 2220	46700 ± 9760
$AUC_{0\text{-}21d}$ of LH, h*mIU/mL	2320 ± 1350	1170 ± 1060	566 ± 509	324 ± 162	3140 ± 1280
$AUC_{0\text{-}21d}$ of FSH, h*mIU/mL	3160 ± 787	2180 ± 716	1570 ± 816	1740 ± 634	2380 ± 430
$AUC_{0\text{-}21d}$ of progesterone, h*nmol/mL	447 ± 708	316 ± 143	278 ± 129	171 ± 76.3	5740 ± 3460

Data are mean ± SD.
